# Assessment of the Predictive Value of Preoperative Serum Albumin and Postoperative Albumin Drop (ΔAlb) for Complications after Pancreas Surgery: A Single-Center Cross-Sectional Study

**DOI:** 10.3390/jcm12030972

**Published:** 2023-01-27

**Authors:** Sérgio Gaspar-Figueiredo, Ismail Labgaa, Nicolas Demartines, Markus Schäfer, Gaëtan-Romain Joliat

**Affiliations:** 1 Department of Visceral Surgery, Lausanne University Hospital CHUV, University of Lausanne (UNIL), 1011 Lausanne, Switzerland; 2 Graduate School for Health Sciences, University of Bern, 3012 Bern, Switzerland

**Keywords:** predictors, morbidity, pancreatectomy

## Abstract

Background: Serum albumin has been shown to be predictive of complications after various gastrointestinal operations. The present study aimed to assess whether preoperative serum albumin and serum albumin drop on postoperative day 1 are associated with postoperative complications after pancreatic surgery. Methods: A single-center cross-sectional study was performed. All patients who underwent pancreatectomy between January 2010 and June 2019 and had preoperative serum albumin value and serum albumin value on postoperative day 1 were included. ΔAlb was defined as the difference between preoperative serum albumin and serum albumin on postoperative day 1. Binary logistic regressions were performed to determine independent predictors of postoperative complications. Results. A total of 185 patients were included. Pancreatoduodenectomies were performed in 133 cases, left pancreatectomies in 36, and other pancreas operations in 16. The preoperative serum albumin value was found to be an independent predictor of complications (OR 0.9, 95%CI 0.9–1.0, *p* = 0.041), whereas ΔAlb was not significantly associated with postoperative complications (OR 1.0, 95%CI 0.9–1.1, *p* = 0.787). The threshold of 44.5 g/L for preoperative albumin level was found to have the highest combined sensitivity and specificity based on the maximum Youden index. Patients with preoperative albumin < 44.5 g/L had a higher incidence of postoperative complications and higher median comprehensive complication index than patients with preoperative albumin ≥ 44.5 g/L. Conclusions: This study highlighted that preoperative serum albumin is an independent predictor of postoperative complications after pancreas surgery.

## 1. Introduction

Pancreatic surgery is a major surgery with high complexity. Due to technical and perioperative improvements, postoperative mortality has decreased over recent decades from 40–50% in the 1950s to <5% in the late 1990s [1,2,3]. This impressive improvement has not been achieved in postoperative morbidity, which remains between 40 and 70% [4,5]. The most frequent and worrisome complications after pancreatic surgery are pancreatic fistula, delayed gastric emptying, surgical-site infection, and pancreatic hemorrhage, with important consequences for the patients. Unfortunately, prediction and early detection of postoperative complications after pancreatic surgery remain challenging.

Nowadays, the diagnosis of complications after pancreatectomy is made by clinical judgment, the evolution of plasmatic biomarkers, and radiological imaging [6]. Postoperative lactate increase has been shown to have high sensitivity but poor specificity in predicting postoperative morbidity, and its association with postoperative complications remains complex to interpret [7,8]. A prolonged lactate increase during the first 24 h after pediatric cardiac surgery and abdominal surgery has been associated with postoperative complications [7,8,9,10,11]. C-reactive protein (CRP) is a liver-produced protein, that is a non-specific biomarker of inflammation. Several publications have already demonstrated that the CRP value is a predictive factor of postoperative complications, especially in colorectal surgery, upper-gastrointestinal surgery, and pancreatic surgery with specific thresholds [12,13,14]. Sarcopenia has also been shown to be predictive of complications after pancreatectomy [15,16]. Interestingly, sarcopenia is also associated with a low preoperative serum albumin level [17].

Over the last few years, preoperative serum albumin levels and their evolution during the postoperative course have gained wider interest as a complication predictor in major abdominal surgery. Albumin is produced by the liver and has a half-life of 20 days. Serum albumin rapidly drops in the acute phases of stress. Many factors contribute to this phenomenon, such as product replacement in favor of other acute phase reactants produced in the liver (CRP, fibrinogen), the increased consumption of protein in high-metabolic postoperative states, dilution, or capillary leak [18,19,20]. In addition, albumin is a non-expensive and easy-to-use parameter. Authors have recently started to assess correlations between postoperative albumin serum level drop (or percentage of decrease) and postoperative complications. Hübner et al. in 2016 suggested this hypothesis in a prospective study of 70 patients [21]. Evidence about its use as an early predictor of morbidity is growing, especially in esophageal and liver surgery [22,23,24,25].

The present study aimed to evaluate if preoperative serum albumin and serum albumin drop on postoperative day 1 (ΔAlb) were associated with the occurrence of postoperative complications after pancreatic surgery.

## 2. Methods

### 2.1. Patients and Eligibility Criteria

All patients with pancreatectomy between 1 January 2010 and 30 June 2019 in the Department of Visceral Surgery of Lausanne University Hospital (CHUV) were eligible. All types of open or laparoscopic surgeries were included (pancreatoduodenectomy, distal pancreatectomy, total pancreatectomy, necrosectomy, or ampullectomy). Inclusion criteria were age > 18 years old, recent (<30 days) preoperative serum albumin value, and serum albumin value on the first postoperative day. Patients who did not consent or who had concomitant surgery in addition to pancreatic resection were excluded.

### 2.2. Operative Procedures and Perioperative Management

Since 2012, enhanced recovery after surgery (ERAS) pathways have been implemented for pancreatic surgery, which increased the standardization of perioperative management. Regarding pancreatoduodenectomy, the non-pylorus preserving technique was performed with omega reconstruction. The technique and type of pancreatic anastomosis was surgeon-dependent, but pancreaticojejunostomy was routinely performed in the case of a large main pancreatic duct (>3 mm) and hard pancreas texture. Amylase and lipase levels in the drain were measured on postoperative days 3 and 5 for pancreatoduodenectomy and distal pancreatectomy. Spleen resection was added to distal pancreatectomy in case of cancer.

### 2.3. Complications

Clavien classification was used for complication grading [26]. Complications were defined as any deviation from the normal postoperative course. They were collected until postoperative day 90. The comprehensive complication index (CCI, 0–100) was also calculated for each patient [27]. Definitions of delayed gastric emptying (DGE), postoperative pancreatic fistula (POPF), and hemorrhage were based on the classifications of the International Study Group for Pancreas Surgery [28,29,30]. Surgical-site infection (SSI) was defined according to the Centers for Disease Control and Prevention [31]. 

### 2.4. Endpoints

The primary endpoint was the occurrence of complications and their association with the preoperative albumin level. The hypothesis was that a low preoperative serum albumin level was correlated with the development of postoperative complications. 

Secondary endpoints evaluated the relationship between preoperative albumin and CCI, the association between ΔAlb and complications (using CCI and Clavien classification), and the association between preoperative albumin/ΔAlb and readmission rate.

Absolute values of albumin (g/L, N [35–52]) were recorded, and the difference between preoperative albumin and postoperative albumin on postoperative day 1 (ΔAlb) was calculated. The formula was [(preoperative albumin) − (albumin on postoperative day 1) = ΔAlb].

### 2.5. Statistical Analysis

Chi-square tests were used for the comparison of qualitative variables, and according to the normality of variables, Student *t*-tests or Mann–Whitney U tests were used for continuous values. 

Statistical significance was defined as *p*-value < 0.05. The maximum Youden index was used to find the best threshold predictive of complication. Multivariable analysis using binary logistic regression was performed to find predictive factors of complications.

All statistical analyses were performed using SPSS Statistics for Mac, version 26 (IBM Corp., Armonk, NY, USA).

## 3. Results

### 3.1. Patients

A total of 185 patients were included: 84 women (45%) and 101 men (55%). The median age was 67 years (IQR 59–75). Pancreatoduodenectomies were performed in one hundred and thirty-three cases, left pancreatectomies in thirty-six, pancreatectomies with duodenal preservation/necrosectomies in seven, ampullectomies in six, and total pancreatectomies in three. The etiologies of the included patients (*n* = 185) were as follows: periampullary tumors (*n* = 145), neuroendocrine tumors/intraductal papillary and mucinous neoplasms/cystic tumors/gastrointestinal stromal tumors (*n* = 15), chronic pancreatitis (*n* = 10), ductal adenocarcinoma of the tail of the pancreas (*n* = 9), and various (*n* = 6, duodenal ulcer, metastases, lymphoma, retroperitoneal liposarcoma, and splenic epidermoid cyst). Postoperative complications occurred in 135 patients (73%), and 90-day mortality was 4% (8/185). Major complications (≥grade IIIa) occurred in 69 patients (37%), and the median CCI was 22.6 (IQR 8.7–34.6).

### 3.2. Serum Albumin and Complications

The median preoperative albumin and ΔAlb of the entire cohort were 41 g/L (IQR 36–44) and 12 g/L (IQR 9–14), respectively. Median preoperative albumin levels and ΔAlb were similar in patients with AJCC stages I-II and AJCC stages III-IV (preoperative albumin: 40 g/L, IQR 34–43 vs. 40 g/L, IQR 36–44, *p* = 0.349; ΔAlb: 13 g/L, IQR 9–16 vs. 12 g/L, IQR 10–14, *p* = 0.524). Patients with malignant etiologies had a higher median ΔAlb compared to patients with benign etiologies (12 g/L, IQR 9–15 vs. 9 g/L, IQR 3–13, *p* = 0.03), while no difference was found concerning preoperative albumin levels (41 g/L, IQR 36–44 vs. 42 g/L, IQR 35–43, *p* = 0.912).

Demographics, intraoperative details, and the length of stay (LoS) in patients with and without complications are summarized in Table 1. The median LoS was significantly longer in patients with complications (8, IQR 7–14 vs. 19 days, IQR 14–27, *p* < 0.001). Patients with complications had lower median preoperative albumin compared to patients without complications (40, IQR 36–43 vs. 42 g/L, IQR 38–46, *p* = 0.014), whereas the median ΔAlb was similar in both groups (12, IQR 8–15 vs. 12 g/L, IQR 9–14, *p* = 0.967). The median preoperative albumin was lower in patients with major complications (grade IIIa or higher) compared to patients without major complications (39, IQR 34–43 vs. 42 g/L, IQR 37–44, *p* = 0.005). There was no difference in median ΔAlb between the groups with and without major complications (7, IQR 12–15 vs. 9 g/L, IQR 12–14, *p* = 0.805).

On multivariable analysis (Table 2), preoperative albumin was the only independent predictor of overall complications after pancreas surgery (OR 0.9, 95%CI 0.9–1.0, *p* = 0.041). Conversely, ΔAlb was not found to be predictive of postoperative complications (OR 1.0, 95%CI 0.9–1.1, *p* = 0.787). 

**Figure 1:**
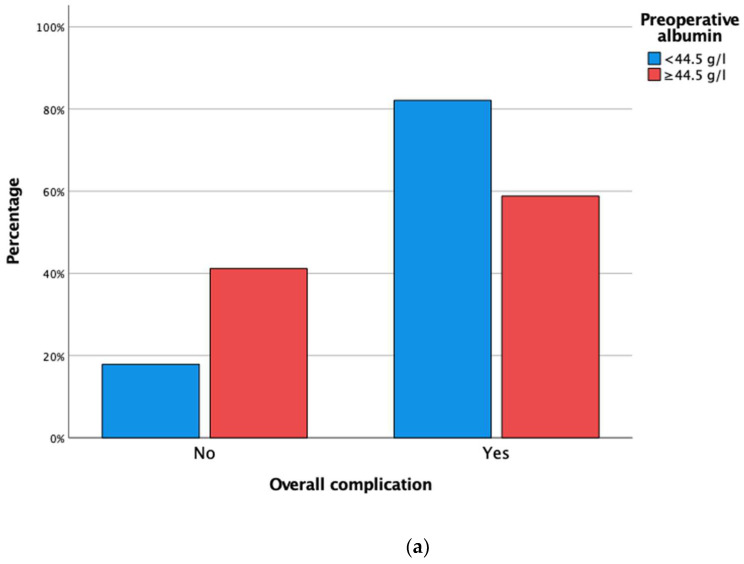

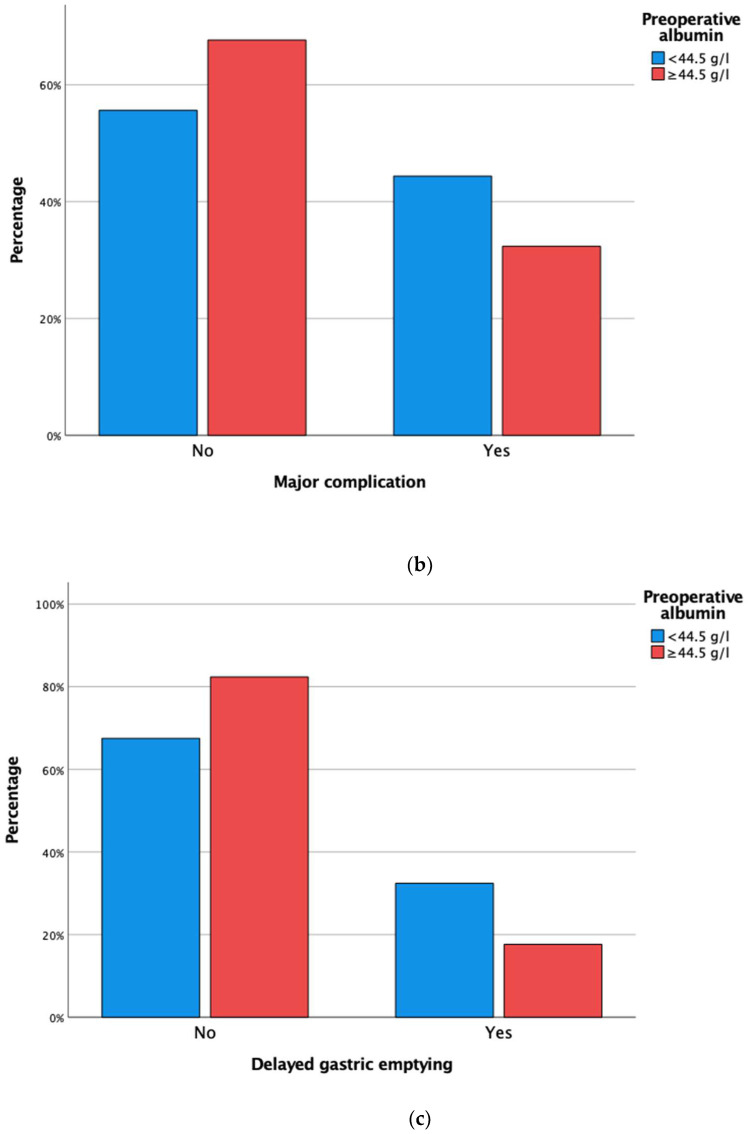

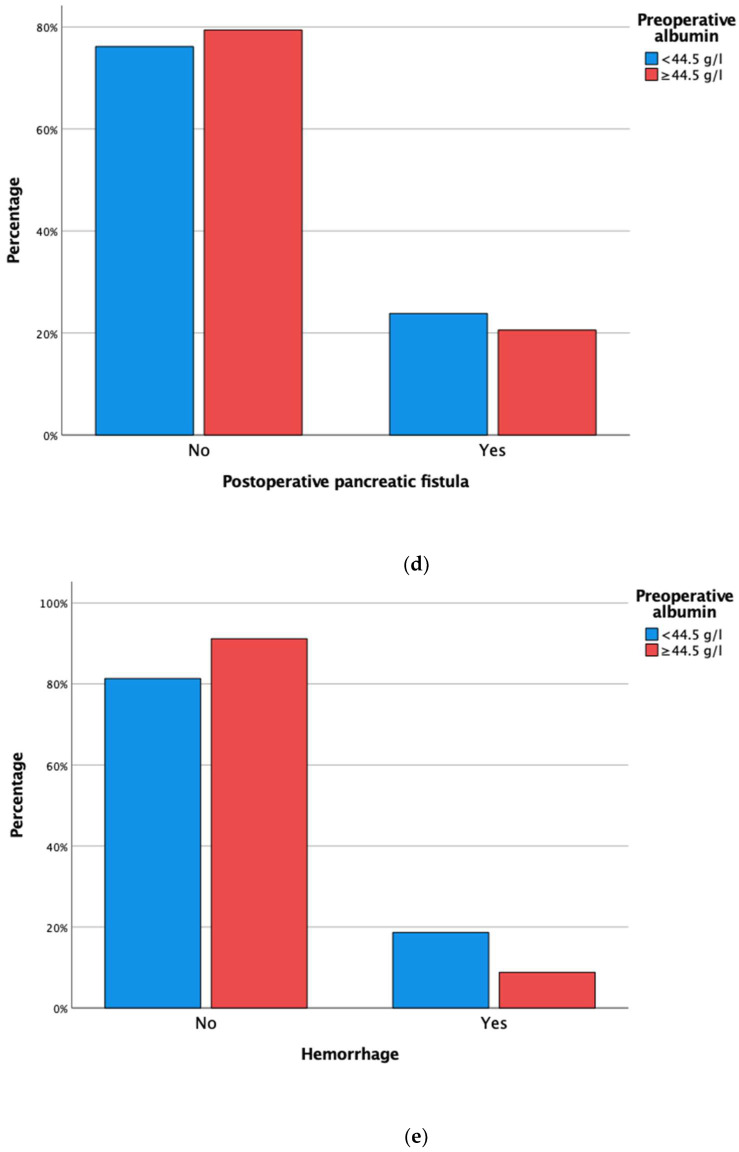

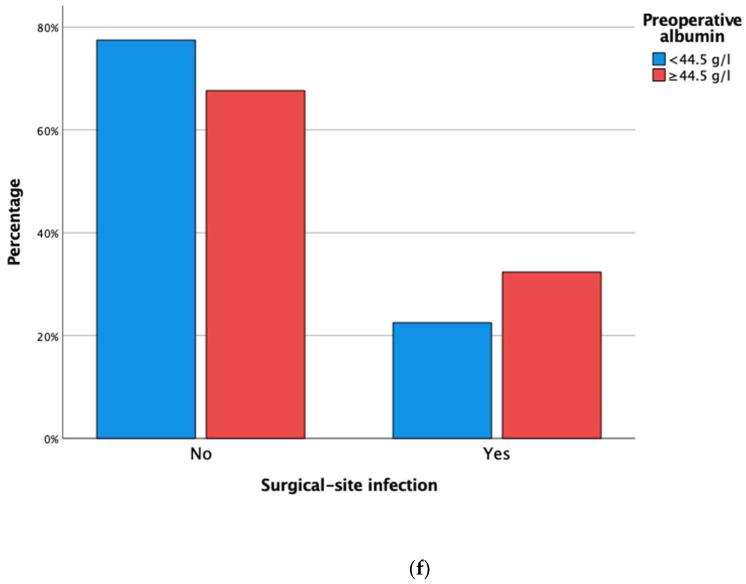
Clustered bar charts for overall complication, major complication, delayed gastric emptying, postoperative pancreatic fistula, hemorrhage, and surgical site infection comparing patients based on preoperative serum albumin value. (**a**) Overall complication (*p* = 0.011); (**b**) Major complications (*p* = 0.250); (**c**) Delayed gastric emptying (*p* = 0.1); (**d**) Postoperative pancreatic fistula (*p* = 0.824); (**e**) Hemorrhage (*p* = 0.210); (**f**) Surgical-site infection (*p* = 0.507).

The area under the ROC curve for preoperative albumin to discriminate for overall complications was 0.63. The cut-off for preoperative albumin with maximal sensitivity and specificity for complication based on the highest Youden index was 44.5 g/L (sensitivity 67%, specificity 86%). When separated into two groups based on this value, patients with preoperative albumin < 44.5 g/L had more overall complications (82% vs. 59%, *p* = 0.011, Figure 1a). No difference in terms of major complications was observed between the groups with preoperative albumin < 44.5 g/L and ≥44.5 g/L (44% vs. 32%, *p* = 0.250, Figure 1b). Patients with preoperative albumin < 44.5 g/L had a higher median CCI compared to patients with preoperative albumin ≥ 44.5 g/L (27.6, IQR 20.9–42.7 vs. 20.8, IQR 0–26.6, *p* = 0.002). No difference in terms of DGE, POPF, hemorrhage, and SSI was found between patients with preoperative albumin < 44.5 g/L and ≥44.5 g/L (33% vs. 18%, *p* = 0.1, 21% vs. 24%, *p* = 0.824, 19% vs. 9%, *p* = 0.210, and 23% vs. 29%, *p* = 0.507, Figure 1c–f). Moreover, the median LoS was longer in patients with preoperative albumin < 44.5 g/L (17, IQR 12–25 vs. 11 days, IQR 7–21, *p* < 0.001).

### 3.3. Readmission

Patients who were readmitted to the hospital within 90 postoperative days had similar median levels for preoperative albumin and similar median ΔAlb as patients without readmission (40, IQR 34–43 vs. 41 g/L, IQR 36–44, *p* = 0.345 and 10, IQR 4–10 vs. 12 g/L, IQR 9–14, *p* = 0.147). On multivariable analysis, preoperative albumin and ΔAlb were not found as predictors for readmission (OR 1.0, 95%CI 0.8–1.1, *p* = 0.594 and OR 0.9, 95%CI 0.8–1.0, *p* = 0.208).

## 4. Discussion

The main finding of this cross-sectional study was that patients with lower preoperative serum albumin levels developed more complications than patients with higher serum albumin levels. Moreover, on multivariable analysis, preoperative serum albumin was found to be an independent factor predictive of postoperative complications after pancreas surgery.

Interestingly, preoperative albumin was found to be associated with the occurrence of postoperative complications. Serum albumin represents, in this case, a surrogate marker influenced by several conditions, such as the nutritional status and inflammatory state. Several articles have demonstrated the relationship between low preoperative albumin and impaired nutritional status [32,33]. Additionally, it has been shown that sarcopenia correlated with lower preoperative albumin levels in pancreatoduodenectomy patients [17]. Moreover, potential dehydration and liver function have also been shown to modulate serum albumin levels [14]. A threshold of 44.5 g/L, which was calculated using the highest Youden index, was found to be the most discriminative in this cohort, with good sensitivity (67%) and specificity (86%). The discriminative power of preoperative albumin based on the area under the ROC curve was judged to be moderate or intermediate (AUC: 0.63).

Unlike descriptions in other reports, in the present study, postoperative albumin drop (ΔAlb) was not found to be associated with postoperative complications. Other studies have shown that ΔAlb is associated with postoperative complications in several types of abdominal operations [22,23,24,25]. Regarding pancreas surgery, a recent systematic review displayed one study that assessed the predictive role of ΔAlb for long-term outcomes [25]. This study found an association between ΔAlb and disease-free survival but did not evaluate its predictive role for complications [34]. The perioperative variations of albumin levels are linked to multiple phenomena, such as a capillary leak, blood dilution, intra-, and postoperative fluid management, or production decrease. It is interesting that ΔAlb was not predictive of complications in this cohort of pancreas surgery patients, which could be due to the specificities of pancreas surgery or due to particular characteristics of the study patients. Further large-scale research investigating ΔAlb after pancreas surgery is mandatory to confirm the present findings.

The results of this study may have clinical implications. As preoperative albumin was associated with complications and as it has been established that the albumin level was correlated with nutritional status, patients with lower preoperative serum albumin value may be good candidates for preoperative nutritional support using oral supplements or even enteral nutrition. It would also be interesting to test if intravenous albumin administration in patients with low preoperative albumin levels could decrease the postoperative complication rates. Moreover, preoperative serum albumin may also be used to help tailor the immediate postoperative follow-up (e.g., early CT scan for patients with low preoperative albumin and high CRP on a specific postoperative day).

Limitations of the present study have to be mentioned. The retrospective design may have induced collection and secular biases. Additionally, the monocentric of this study impairs the generalizability of the results. Potential selection bias may have occurred as not all patients with pancreatectomy had a preoperative albumin value or albumin value on postoperative day 1. The heterogeneity of the cohort regarding surgical indications, types of surgery, and postoperative courses depending on the surgical procedures is also a limitation of this study.

In summary, this study suggests that patients with lower preoperative serum albumin levels developed more postoperative complications and identified preoperative serum albumin as an independent predictor of overall morbidity after pancreas surgery.

## Figures and Tables

**Table 1:** Demographics, intraoperative details, and length of stay for patients with and without complications.

	Complications (*n* = 144)	No Complications (*n* = 41)	*p*-Value
Age, years	70 (60–77)	68 (57–74)	0.308
Sex, women/men	63/81	21/20	0.397
BMI, kg/m^2^	25 (23–29)	25 (22–27)	0.644
Active smokers	36	11	0.812
Diabetes mellitus	25	5	0.429
Jaundice	80	23	0.951
Preoperative stenting	74	19	0.567
ASA score	5/89/46/4	3/28/10/0	0.599
Neoadjuvant treatment	8	2	0.866
PDAC	96	25	0.499
Whipple, distal, others	108/26/10	26/10/5	0.311
Portal vein resection	37	11	0.884
Operative time, minutes	336 (286–409)	329 (239–390)	0.225
Blood loss, ml	500 (200–800)	350 (100–650)	0.095
Length of stay, days	19 (14–27)	8 (7–14)	<0.001

BMI—body-mass index, ASA—American Society of Anesthesiologists, PDAC—pancreatic ductal adenocarcinoma. Patients with postoperative death (*n* = 10) were included in the group with complication.

**Table 2:** Uni- and multivariable logistic regression for factors predictive of postoperative complications after pancreatectomy.

	Univariable OR (95% CI)	*p*-Value	Multivariable OR (95% CI)	*p*-Value
Age, years	1.0 (1.0–1.0)	0.247		
BMI, kg/m^2^	1.0 (0.9–1.1)	0.667		
Active smokers	0.9 (0.4–2.0)	0.767		
Diabetes mellitus	1.4 (0.5–3.9)	0.550		
Preoperative stenting	1.2 (0.6–2.3)	0.679		
ASA score, I-II/III-IV	1.1 (0.5–2.3)	0.817		
Operative time, min	1.0 (1.0–1.0)	0.372		
Blood loss, ml	1.0 (1.0–1.0)	0.177	1.0 (1.0–1.0)	0.295
Preoperative albumin, g/L	0.9 (0.9–1.0)	0.013	0.9 (0.9–1.0)	0.041
ΔAlb, g/L	1.0 (0.9–1.1)	0.787		

BMI—body-mass index, ASA—American Society of Anesthesiologists, Alb—preoperative albumin-albumin on postoperative day 1, OR—odds ratio.

## Data Availability

The data presented in this study are available on request from the corresponding author.
